# Physiological, molecular and histological response of *Pentodon bispinosus* larvae (Coleoptera: Scarabaeidae) as a novel bioindicator for soil pollutants

**DOI:** 10.1038/s41598-025-15417-7

**Published:** 2025-08-25

**Authors:** Hanaa S. Hussein, Toqa Abdel Nasser, Marwa Saad, Ahmed S. Abo-Shanab, Lamia M. El-Samad

**Affiliations:** 1 Department of Applied Entomology and Zoology, Faculty of Agriculture, Alexandria University, Alexandria, 21545 Egypt; 2 Department of Zoology, Faculty of Science, Alexandria University, Baghdad st., Qism Moharram Bek, Alexandria, 21568 Egypt; 3 Central Agricultural Pesticides Laboratory, Agricultural Research Center, Giza, Egypt

**Keywords:** *Pentodon bispinosus*, Bioindicator, Molecular damage, Cell death, Polluted soil, stress markers, Ultrastructural analysis, Biochemistry, Cell biology, Physiology, Zoology, Environmental sciences

## Abstract

This study investigates the impact of soil pollution exposure on the physiological and cellular homeostasis of *Pentodon bispinosus* larvae. The beetle larvae were collected from two distinct environments: an organic site (control) and a chemical-treated site (insecticides & mineral fertilizers). The larvae were subjected to physiological, biochemical, histological, and ultrastructural analyses to evaluate stress markers, DNA damage, cell viability, and morphological changes. SEM-EDX microanalysis revealed elemental variations in larval midgut from treated soil. Apoptosis and necrosis were measured, a significant differences in cell viability observed between the polluted and control groups. Comet assay was utilized to assess DNA damage, showing increased genotoxic effects in the treated group. Biochemical assays for oxidative stress markers, including ALT, AST, MDA, SOD, CAT, GPx, and APOX, indicated a higher oxidative burden in larvae from polluted soil. Statistical analysis revealed significant physiological and biochemical disruptions in the treated larvae, supporting the hypothesis that soil pollution exposure adversely affects cellular and molecular integrity, leading to potential disturbances in homeostasis. Histological and ultrastructural examinations demonstrated cellular disruptions in the larvae exposed to polluted soil. This research unveils and highlights the novel impacts of soil pollution on the larvae of white grub beetle, with implications for ecosystem health, and the potential use of these larvae as a promising assessment bioindicator for soil pollution.

## Introduction

The potato, *Solanum tuberosum* L. (Family Solanaceae), is an annual dicotyledonous tuber crop that has expanded globally^1^. Potatoes are the world’s fourth most produced food crop, following wheat, maize, and rice, and the principal non-cereal crop. Potato is one of Egypt’s most important food crops. Potatoes are a crucial staple meal known for their nutritional value, which includes proteins, carbohydrates, vitamins, and minerals^2^. Their extraordinary production potential outperforms many other crops, making them contributes to Sustainable Development Goals (SDGs), including zero hunger, sustainable agriculture, and economic opportunity^1^, . According to FAO^3^, potatoes are a staple crop for billions of people. Potatoes are an important crop across agrifood systems globally, ranging from smallholders cultivating numerous heritage varieties by hand in the Andes to large commercial, automated farms in different continents.

Despite the use of pesticides and non-chemical treatments, it is estimated that 37% of all crops are lost each year to pests, 13% due to insects^4^. The use of pesticides in potato production results in the buildup of Fe, Pb, Cd, Cr, and Zn metals in the soil, particularly in the rooting zone. Increased metal concentration in soil has a direct influence on environmental pollution and an indirect impact on plant, animal, and human health^5,6^. Mineral fertilizers use is a global issue, such fertilizers are utilized to increase agricultural yields. These fertilizers include both heavy metals and vital mineral components^7^. Heavy metals (HMs) contamination is becoming a significant issue in the farming ecology, causing substantial health consequences for many living organisms. Cobalt (Co), zinc (Zn), iron (Fe), copper (Cu), nickel (Ni), and manganese (Mn) are examples of heavy metals (HMs) that are necessary for normal development, electron exchanges, and other critical metabolic functions. Other heavy metals (HMs) on the other hand, such as As, Cd, Cr, and Pb, have no biologically useful role and can be extremely harmful even at low concentrations. Orellana-Mendoza, et al., and Zhang, et al.^7,8^ stated that heavy metals in soil come from both natural and human sources; heavy metals naturally occur in soils in trace amounts because of rock weathering processes. However, their concentration rises owing to human activities like mining, smelting, and the use of fertilizers and pesticides.

In recent decades, there has been a significant increase in awareness of detrimental environmental change worldwide. Bioindication is not a traditional conception; rather, it has become a promising new issue concerning assessing environmental conservation. Bioindicator refers to a species that detect the environment’s abiotic or biotic status. It demonstrates how environmental changes such as high metal concentrations impact a community or ecological system, habitat and whether that change has a beneficial or harmful impact. Beetles (Coleoptera) are the most diverse group of insects, with 300,000-450,000 identified species^9^. They are easily sampled using several ways and have previously proposed as bioindicators in numerous studies^10,11^. Tőzsér, et al.^12^ suggested that the decontamination and ground beetle excretion of pollutants are effective in low or moderately contaminated environments, while disposal is inefficient in extremely high contaminated areas, causative to intense accumulation of pollutants in ground beetles. Coleopterans, particularly the Scarabaeidae, Carabidae, Staphylinidae, and Curculionidae, are thought to represent potential bioindicators. The ground beetle is the most common insect used in environmental biomonitoring, notably for assessing environmental contaminants^13^.

In the field, potatoes are attacked by a variety of insects, such as potato tuber moth, *Phthorimaea operculella* (Zeller) (Or.: Lepidoptera: Fam.: Gelechiidae), the cotton leafworm, *Spodoptera littoralis* (Boisd.) (Or.: Lepidoptera: Fam.: Noctuidae) and the white grub beetle, *P. bispinosus* (Kuster) (Or.: Coleoptera: Fam.: Scarabaeidae)^14^.

Many scarab beetles spend most of their lives underground as larvae, feeding on roots, causing damage to crops, especially tuber crops like potatoes. The white grub, *P. bispinosus* (Kuster), is a major root-feeding insect^15^. White grubs are a major problem in Egypt’s potato crop. The first larval instar consume organic material, whereas the second and third instar larvae move beneath the soil to consume roots or tubers^16^. Third-instar larvae cause tuber damage^17^. White grubs can damage tubers and lower crop quality by infecting them with a variety of bacterial and fungal pathogens^18^.

The objectives of the present study were to inspect the physiological, molecular, biochemical, and histological impacts of soil contamination with heavy metals on midgut tissues of *P. bispinosus* larvae, and to prove that white grub beetles are promising bioindicators for soil pollution.

## Materials and methods

### Hypothesis

This study examines how soil pollution affects homeostasis in *P. bispinosus* larvae by comparing individuals from an organic site and a chemically treated site. It evaluates stress marker levels, molecular damage (to lipids, and DNA), cell death rates (apoptosis and necrosis), and structural changes in the midgut. The research aims to reveal significant cellular responses to pollution, highlighting the impact of heavy metals on beetle larvae from organic and polluted environments.

### Study area and sample collection

**Fig. 1:**
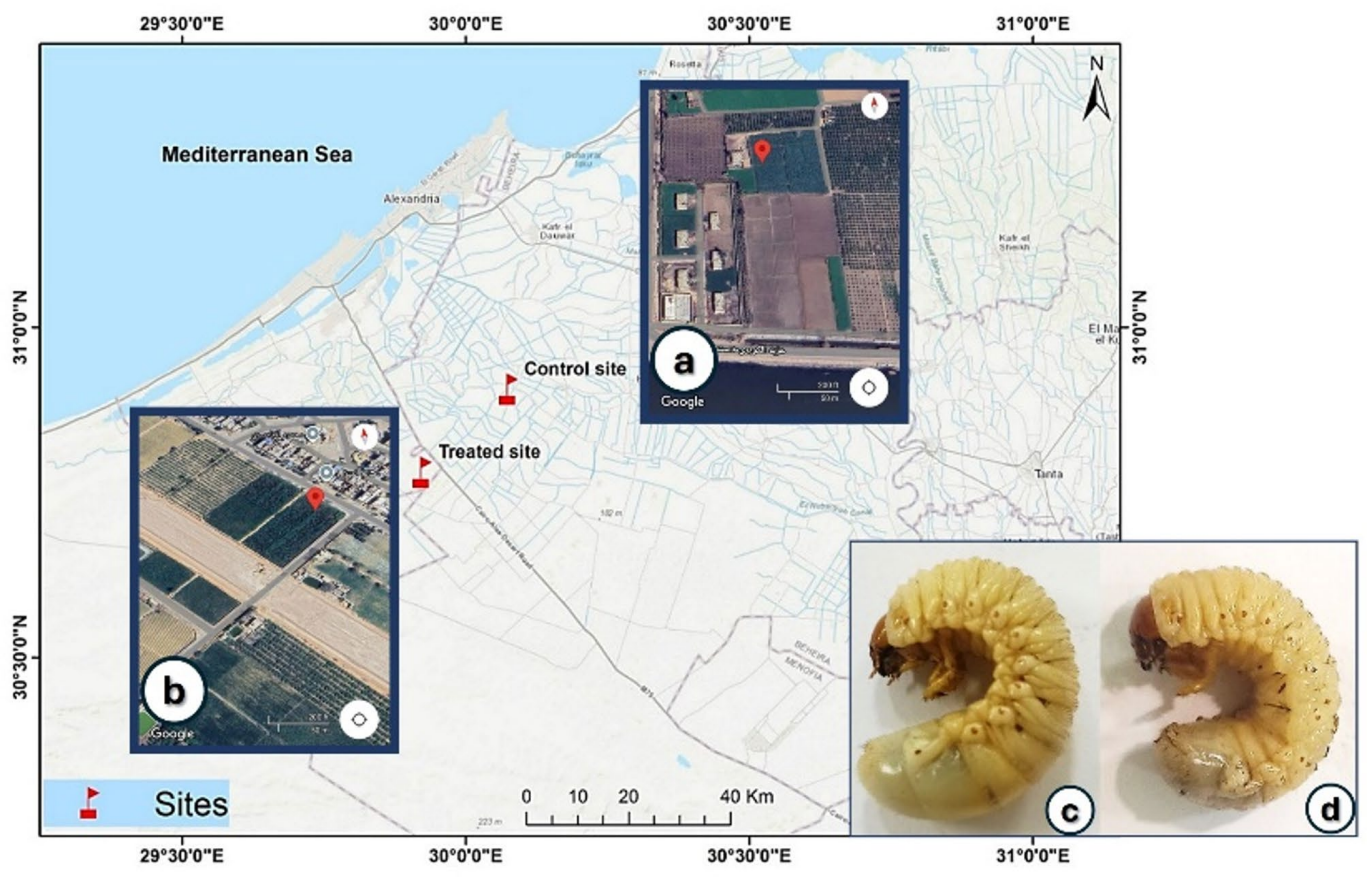
Map of El-Beheira Governorate, (**a**) (control site) (30° 48′ 39.3″ N 30° 17′ 41.8″ E) and (**b**) (polluted site) (30° 40′ 01.3″ N 30° 03′ 48.9″ E). (**c**) *P bispinosus* larvae from control site, (**d**) larvae from polluted (treated) site, Source: ESRI, Maxar, Earthstar Geographics, and the GIS user. The maps were extracted by using a version of Esri’s Geographic Information System (GIS) software (ArcGIS 10.5).

The study involved collecting *P. bispinosus* third instar larvae of approximately the same size range from two sites in El-Beheira Governorate, Egypt: Control site (A), an organic farm the owner doesn’t use any chemicals, (R75V + XHQ Housh Esa) (30°48′ 39.3″N 30°17′41.8″E) and polluted site (B), a commercial farm that use chemicals (insecticides & fertilizers), (M3C8 + 2QH West EL-Nouparia) (30°40′ 01.3″N 30°03′48.9″E) (Fig. 1). Larvae were manually gathered and transported to the Faculty of Agriculture, Alexandria University, where they were kept in plastic containers filled with 6 cm of organic matter from their collection sites, then the larvae were used for further analysis.

### Experimental design

This study evaluated the effects of heavy metal-contaminated soil on the morphology, physiology, and biochemical responses of *P. bispinosus* larvae. Larvae were collected from two environments: an organic farm (control) and a chemically treated commercial farm (polluted). A comparative approach was used, with all larvae undergoing the same acclimation process under laboratory conditions before dissection and analysis. The study assessed midgut morphological, histological, and cellular changes, as well as enzymatic activity levels, including antioxidant enzymes (SOD, CAT, GR, APOX, GPx), MDA, GSH, cytochrome P450, and AChE. Experimental procedures were standardized to reduce variability, and statistical analyses were conducted to identify significant differences between control and polluted groups.

### X-ray detection in the midgut of *P. bispinosus* larvae

Elemental analysis of the *P. bispinosus* larval midgut was performed using scanning microscope - energy-dispersive X-ray microanalysis (SEM-EDX) microanalysis (JEOL JSM-5300) at the Faculty of Science, Alexandria University. Four larvae from each group (control and polluted) were randomly selected. After being anesthetized on ice, their midguts were dissected and prepared for microscopic examination. The tissues were frozen at − 70 °C, lyophilized at − 35 °C for 8 h, and mounted on aluminum stubs with carbon adhesive tape. To protect the samples during SEM exposure and ensure accurate analysis, the surfaces were coated with a gold film (≥ 20 nm)^19^. The prepared tissue slides, covering a 500 μm diameter area, were analyzed using SEM-EDX in stationary spot mode at 500× magnification for 110 s. The SEM-EDX software automatically identified elemental peaks and measured the signal intensity of each element present in the midgut samples. Calibration standards with known compositions were used to ensure accurate comparison and quantification of elemental concentrations^20^. The SEM-EDX analysis parameters are detailed. Randomly selected tissue sections were analyzed to detect elemental distributions, with results visualized and compared across groups. Representative spectra and elemental maps are providing qualitative and quantitative insights into the elemental composition of the samples^21^.

### Evaluation of cell viability and DNA damage

#### Cell viability assay

Apoptotic cells were detected using the Annexin V-FITC Apoptosis Detection Kit (R&D Systems, USA), which identifies early apoptosis by binding Annexin V-FITC to externalized phosphatidylserine. Cell suspensions (1 × 10⁵ to 1 × 10⁶) were washed with cold calcium-containing PBS, then incubated in a reagent mix containing binding buffer, Annexin V-FITC, and propidium iodide for 15 min in the dark at room temperature. After washing and resuspension, cells were analyzed by flow cytometry (FL1 and FL2 channels), and classified as viable, apoptotic, or necrotic based on fluorescence, using untreated controls for gating Koopman, et al. and Kamiloglu, et al.^22,23^. Additionally, to perform molecular analyses on midgut, four individuals were sampled from both the control and polluted groups. Cell viability was evaluated using the Annexin V-FITC and propidium iodide (PI) staining assay kit (Sigma–Aldrich, Germany). Midgut tissues homogenate were prepared in a cold buffer (PBS) (pH 7.4; 4 °C) as per Koopman, et al. and Kamiloglu, et al.^22,23^. Cell suspensions were washed twice with PBS, resuspended in 195 µL of binding buffer, and incubated with 5 µL of Annexin V-FITC in the dark for 15 min. After another wash, cells were resuspended in 190 µL of binding buffer and mixed with 10 µL of propidium iodide (PI) solution. Samples were immediately analyzed by flow cytometry (Becton Dickinson, USA), and fluorescence data were processed using Cell Quest Pro software (version 5.2.1) to classify cells as viable, early apoptotic, late apoptotic, or necrotic.

#### DNA damage

DNA damage in midgut cells was assessed using the alkaline comet assay, following the method of^24^. Midgut tissues were gently macerated in cold Hank’s balanced salt solution containing 20 mM EDTA and 10% DMSO. The suspension was centrifuged (10 min, 500×g, 4 °C), and the pellet was resuspended in 0.5 mL PBS. Slides pre-coated with 0.65% normal-melting point agarose (NMPA) were used to spread a mixture of cell suspension and 0.65% low-melting point agarose (LMPA, ~ 40 °C). After solidification at 4 °C, a third layer of LMPA was added. Slides were then lysed overnight at 4 °C in a lysis buffer (pH 10.0), washed, and immersed in alkaline electrophoresis buffer (pH 13.0) for 20 min at 4 °C. Electrophoresis was performed for 20 min at 300 mA. Slides were then neutralized in Tris buffer, dehydrated with ethanol, rehydrated, and stained with ethidium bromide (20 µg/mL). Comet images were visualized using a Leitz Orthoplan epifluorescence microscope, and 100 comets per slide were analyzed, with three replicates per group.

### Biochemical assays

Larval midgut tissues were dissected on ice, rinsed with 0.9% saline, blotted dry, weighed, and homogenized in Tris-HCl buffer (pH 7.4) at a 1:5 ratio. The homogenate was centrifuged at 15,000×g for 60 min at 4 °C, and the supernatant was stored at − 70 °C for later analysis. Biochemical markers, including ALT and AST, and oxidative stress indicators such as MDA, SOD, CAT, GPx, and APOX were measured. ALT and AST activities were assessed using BioSystems S.A. assay kits (REFs M11531i-24 and M11533i-24), following the manufacturer’s instructions with adaptations for insect tissues^25^. For the assays, ALT and AST reagents were mixed with sample supernatants at a 10:1 (v/v) ratio, vortexed for 3 s, and incubated at 37 °C for 30 s. Absorbance was measured at 340 nm using a spectrophotometer, and enzyme activities were expressed as units per milligram of protein (U/mg protein). One unit (U) of ALT or AST activity was defined as the amount of enzyme that catalyzes the conversion of 1 µmol of substrate per minute at 37 °C.Superoxide dismutase (SOD) activity, also expressed as U/mg protein, was determined according to the method described by [author/reference to be inserted^26^. Superoxide dismutase (SOD) activity was measured by monitoring the rate of adrenaline autooxidation. The reaction mixture included sample supernatant, 200 mM sodium carbonate buffer (pH 10.0), 15 mM freshly prepared epinephrine, and 10 mM EDTA. Absorbance changes at 480 nm were recorded, and one unit (U) of SOD activity was defined as the amount of enzyme required to cause 50% inhibition of adrenaline autooxidation under these conditions. Catalase (CAT) activity was measured spectrophotometrically using Aebi^27^ method by monitoring the decrease in hydrogen peroxide (H₂O₂) absorbance at 240 nm. The reaction mixture included sample supernatant, 50 mM phosphate buffer (pH 7.0), and 10 mM H₂O₂. CAT activity, expressed as U/mg protein, was calculated based on the rate of H₂O₂ decomposition. One unit (U) of CAT activity was defined as the amount of enzyme that decomposes 1 µmol of H₂O₂ per minute at 25 °C^28^.

Glutathione (GSH) levels were measured using 5,5’-dithio-bis-2-nitrobenzoic acid (DTNB) as a chromogenic substrate. The reaction mixture included the sample supernatant, GSH diluent (prepared with double-distilled water at a 1:9 ratio), a 1 mM GSH standard solution, and 3.07 mg of GSH dissolved in 10 mL of diluent. After adding 1.5 mL of DTNB, the reaction was initiated, and absorbance was measured at 412 nm. GSH concentrations were then calculated using a standard curve generated from known GSH standards.

GPx enzyme activity, expressed as (U/mg protein), was determined using the method outlined by^29^. GPx activity was measured using cumene hydroperoxide (cumOOH) as a substrate to assess the enzyme’s ability to oxidize 1 µmol of NADPH per minute. The reaction mixture contained sample supernatant, 50 mM potassium phosphate buffer (pH 7.0), 5 mM EDTA, 1 mM sodium azide (to inhibit catalase), 0.25 mM H₂O₂, 1.5 mM cumOOH in ethanol, 2 mM reduced glutathione (GSH), 50 U/mL glutathione reductase (GR), and 0.2 mM NADPH. Absorbance at 340 nm was monitored over 5 min to determine GPx activity. One unit (U) of GPx activity was defined as the amount of enzyme that oxidizes 1 µmol of NADPH per minute at 25 °C.

Ascorbate peroxidase (APOX) activity was measured by monitoring the oxidation of ascorbate in the presence of hydrogen peroxide (H₂O₂). The reaction mixture contained 50 mM phosphate buffer (pH 7.0), 0.5 mM ascorbate, and 1 mM H₂O₂. Tissue samples were homogenized in 50 mM phosphate buffer (pH 7.0) with 1 mM EDTA and centrifuged at 12,000 × g for 20 min to obtain the enzyme extract. The reaction was started by adding the enzyme extract, and the decrease in absorbance at 290 nm was recorded. APOX activity was calculated using the molar extinction coefficient of ascorbate (2.8 × 10⁴ M⁻¹cm⁻¹) and expressed as units per mg protein, where one unit corresponds to the amount of enzyme that oxidizes 1 µmol of ascorbate in one min under assay conditions^30^.

Glutathione Reductase (GR) activity was determined using the method described by^31^. The reaction mixture contained 0.1 M potassium phosphate buffer (pH 7.0), 1 mM EDTA, 0.5 mM NADPH, and 1 mM oxidized glutathione (GSSG). The decrease in absorbance at 340 nm, corresponding to NADPH oxidation, was monitored for 3 min at 25 °C. Enzyme activity was expressed as mU/mg protein One milliunit (mU) of GR activity was defined as the amount of enzyme that catalyzes the oxidation of 1 nmol of NADPH per minute at 25 °C^31^.

Cytochrome P450 (CYP450) enzyme activity was measured in microsomal preparations from the midgut tissue of *Pentodon bispinosus* larvae using p-nitroanisole as the specific substrate. The microsomal fraction was prepared by differential centrifugation (10,000 × g followed by 100,000 × g), and protein concentration was determined using the Bradford assay. Enzyme reactions were carried out in phosphate buffer (pH 7.4) at 30 °C for 30 min, and the reactions were stopped by the addition of cold methanol. The resulting metabolite, p-nitrophenol, was quantified by High-Performance Liquid Chromatography HPLC. CYP450 activity was expressed in milliunits per milligram of protein (mU/mg protein). Control experiments accounted for non-specific activity, and the effects of known inducers and inhibitors (e.g., phenobarbital and SKF-525 A) were also evaluated. This method provides a reliable assessment of CYP450 activity and the factors influencing enzyme function One milliunit (mU) is defined as the amount of enzyme that converts 1 nmol of p-nitroanisole to p-nitrophenol per minute^32^.

Acetylcholinesterase (AChE) activity was measured using acetylthiocholine iodide as the substrate. Microsomal fractions were prepared through homogenization and differential centrifugation, and protein concentration was determined using the Bradford assay. The enzyme reaction was conducted in phosphate buffer (pH 8.0) at 25 °C for 15 min and was stopped by the addition of 5,5’-Dithiobis-(2-nitrobenzoic acid) (DTNB), also known as Ellman’s reagent. The resulting yellow product (5-thio-2-nitrobenzoate) was measured spectrophotometrically at 412 nm. AChE activity was expressed as units per milligram of protein (U/mg protein). Control assays accounted for non-specific activity, and the effects of inhibitors, such as eserine, were evaluated at defined concentrations. One unit (U) of AChE activity was defined as the amount of enzyme that hydrolyzes 1 µmol of acetylthiocholine per minute at 25 °C^33^.

### Histological and ultrastructural analysis

For histological analysis, dissected midgut tissues were fixed in 4% formaldehyde and 1% glutaraldehyde in 0.1 M phosphate buffer (pH 7.2) for 1.5 h at 4 °C. Post-fixation was done with 2% osmium tetroxide in the same buffer at 25 °C for 1.5 h. Samples were washed, then dehydrated at 4 °C through a graded ethanol series (50–100%), each for 15 min, and embedded in an Araldite–Epon resin mixture. Semithin Sect. (1 μm) were cut with a Leica Ultracut UCT25 ultramicrotome, stained with 1% Methylene Blue Borax (Morphisto GmbH, Germany), and examined using an OLYMPUS BX60 light microscope.

For ultrastructural analysis, ultrathin sections were prepared following the standard protocol described by^34^. For histological and ultrastructural analysis, midgut tissues were fixed in 4F1G buffer (pH 7.2), composed of 40% formaldehyde (10 mL), 50% glutaraldehyde (2 mL), monobasic sodium phosphate (1.16 mg), NaOH (0.27 mg), and ultrapure water up to 100 mL. Following fixation, tissues were washed in 0.1 M phosphate buffer for 2 h at 4 °C, then post-fixed in 1% osmium tetroxide (OsO₄) for 2 h at 4 °C. Samples were washed three times in the same buffer (10 min each), dehydrated through a graded ethanol series, treated with propylene oxide, and embedded in an Araldite–Epon resin mixture.

Ultrathin Sect. (70 nm thick) were prepared using an LKB ultramicrotome with a glass knife, mounted on copper grids, and stained with uranyl acetate and lead citrate for transmission electron microscopy (TEM)^35^. Finally, the sections were examined using a JEOL 100CX Electron Microscope operating at 80 kV.

### Statistical procedures

Statistical analysis was conducted using GraphPad Prism 9.4.1. Data normality was assessed using the Shapiro-Wilk test. Unpaired t-tests were used to compare Acetylcholinesterase (AChE) and Cytochrome P450 (CYP450) enzyme activities between treated and untreated groups, as these data were normally distributed. All other traits were analyzed using two-way ANOVA to evaluate the effects of treatment and exposure duration, followed by Tukey’s post hoc tests for multiple comparisons. Results were reported as mean ± SE, and statistical significance was considered at *p* < 0.05. Graphs were generated using GraphPad Prism.

## Results

### Elemental composition in soil and midgut of *P. bispinosus* larvae

Energy-dispersive X-ray microanalysis (EDX) was used to determine heavy metals in soil samples and *P. bispinosus* larval midgut tissues. This analysis was applied using a JEOL (JSM-5300) scanning microscope.

**Fig. 2:**
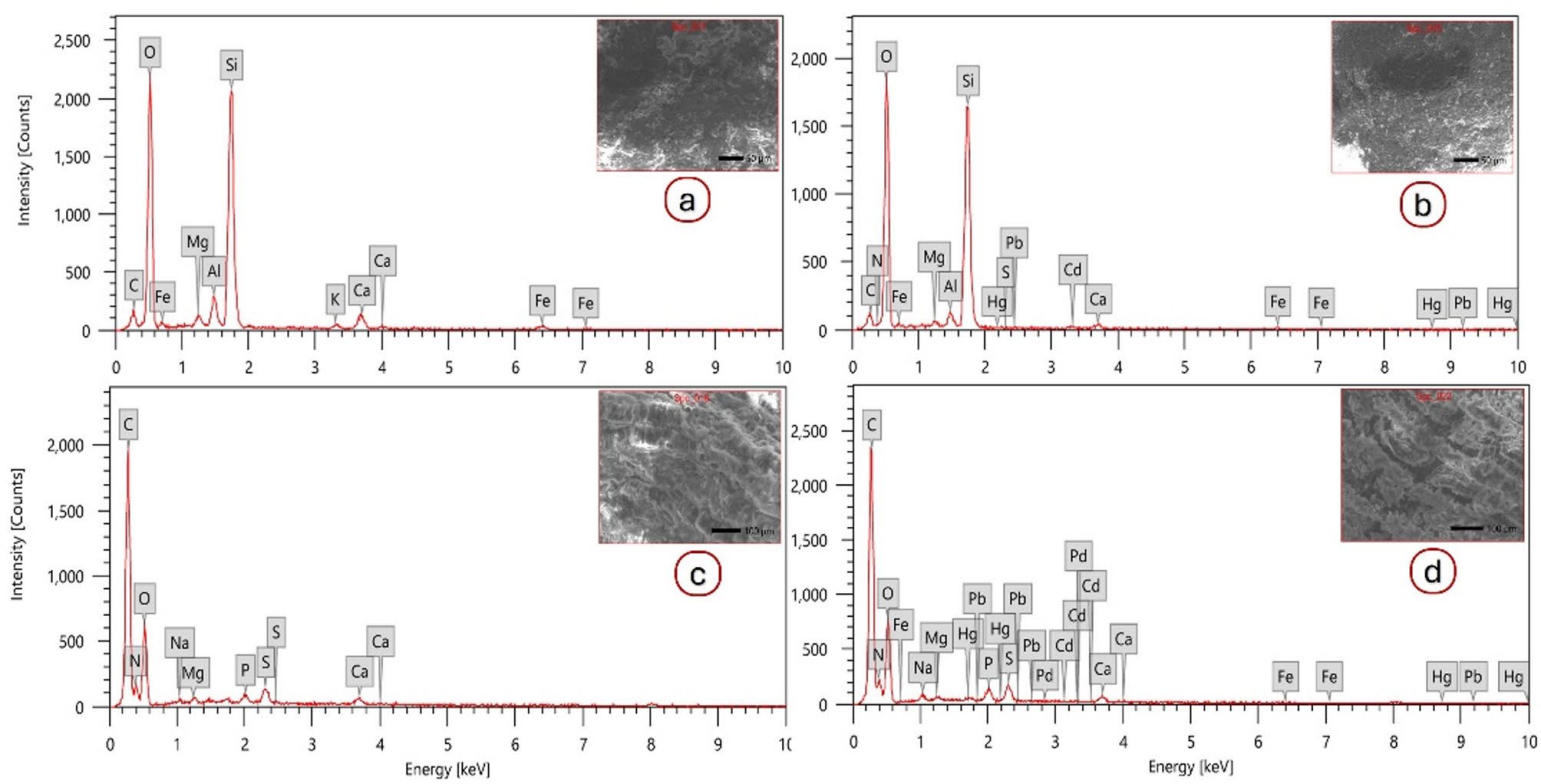
Energy-dispersive X-ray (EDX) spectra of soil samples from selected sites, (**a**) control site and (**b**) polluted site. EDX of the larval midgut, (**c**) from control site and (**d**) from polluted site.

The EDX analysis of soil and *P. bispinosus* larval midgut from control and polluted sites (Fig. 2), showed the presence of some HMs in the soil and larvae taken from the polluted (treated) site compared to their absence in the control samples. The current study exhibited that HMs such as Fe, Cd, Pb, S, N, and Hg showed only within the polluted soil (Fig. 2b) and larvae (Fig. 2d) compared to the control (Fig. 2a & c). That could be due to the accumulation of various mineral fertilizers or the use of different pesticides that cause soil pollution. Due to the excessive uses of pesticides and chemical fertilizers, larvae of ground beetles showed high potential for HMs accumulation. *P. bispinosus* larvae were discovered to be promising indicators of excessive contaminated soil by uptake of heavy metals.

### Assessment of cell viability and DNA damage in the midgut of *P. bispinosus* larvae

**Fig. 3:**
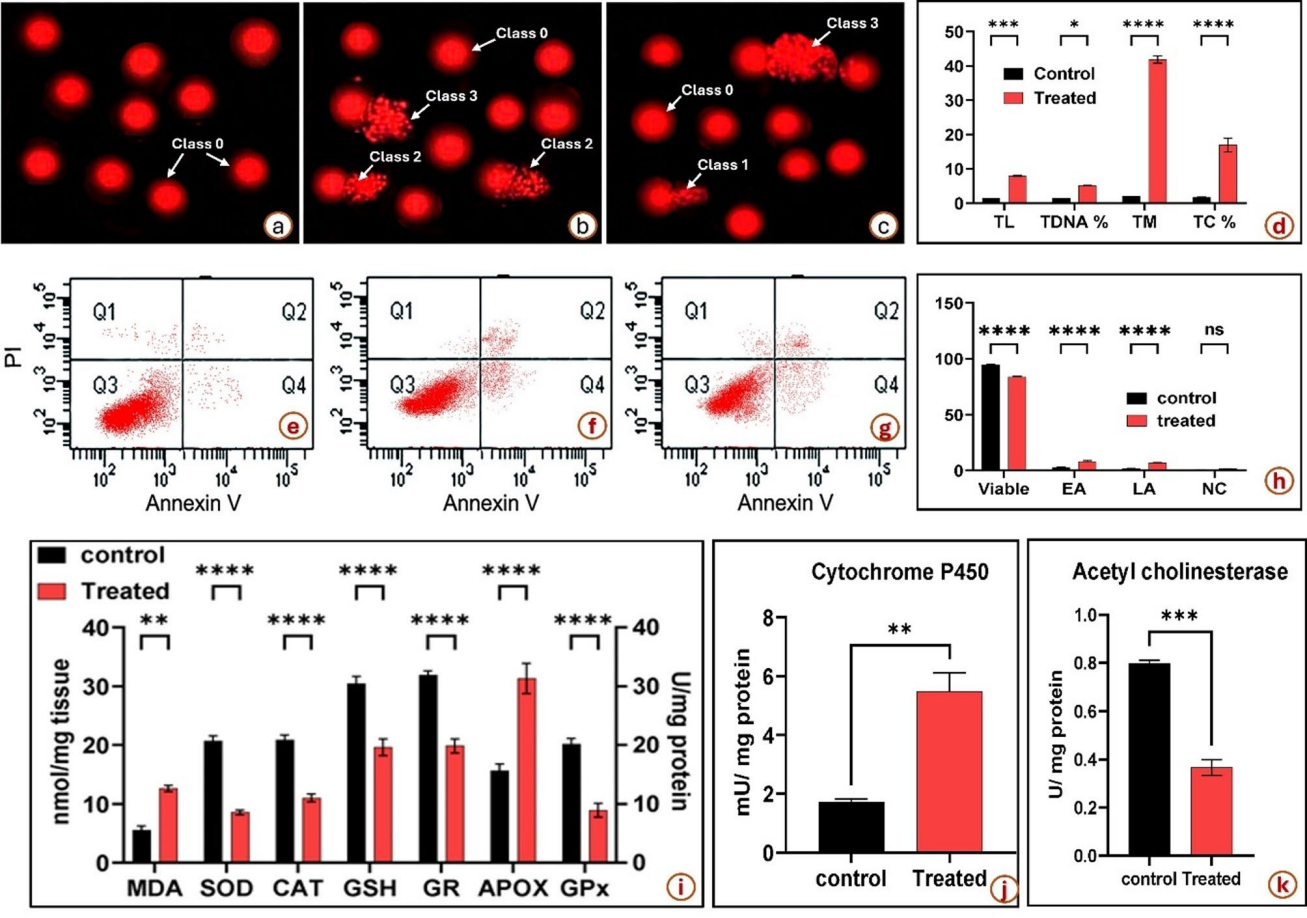
The comet assay showing DNA damage of the midgut tissues of *P. bispinosus* larvae, (**a**) from control site and (**b**, **c**) from polluted site, (**d**) Comet assay parameters, Tail length µm (TL), Tail DNA % (TDNA%), Tail moment (TM), Tailed cell % (TC). Cytometric analysis of annexin V-FITC for beetle midgut, (**e**) from control site and (**f**, **g**) from treated (polluted site), (**h**) the percentages of necrotic Q1, late apoptotic Q2, viable Q3, early apoptotic cell Q4. (**i**) Activities of MDA, GSH, antioxidant enzymes (SOD, CAT, GR, APOX, GPx); (**j**) Activity of Cytochrome P450 (mU/mg protein); (**k**)Activity of AChE, in midgut tissues of *P. bispinosus* from control and treated (polluted) sites. Data are expressed as mean ± SE.

The impact of soil pollution on cellular homeostasis and integrity in *P. bispinosus* larvae was analyzed using Annexin V-FITC and PI staining (Fig. 3). The comparative results between the control group (organic environment) (Fig. 3a) and the treated group (polluted environment) (Fig. 3b &c) revealed significant differences in cellular viability and apoptosis profiles (Fig. 3d). The percentage of viable cells in the control group was significantly higher (94.83%) compared to the polluted or treated group (83.77%). This reduction in viable cells (11.06%) highlights the detrimental effects of exposure to polluted soil on cellular integrity. Early apoptotic cells (EA) constituted 2.593% of the control group and increased to 8.000% in the treated group, indicating a substantial rise in early apoptotic activity (an increase of 5.407%). Similarly, late apoptotic cells (LA) exhibited a marked increase from 1.667% in the control group to 6.833% in the treated group, corresponding to a 5.166% elevation. These results suggest that polluted soil exposure significantly promotes both early and late apoptotic processes in *P. bispinosus* larvae, suggesting that exposure to soil pollution accelerates programmed cell death pathways. The percentage of necrotic cells (NC) showed a modest increase, increasing from 0.9333% in the control group to 1.567% in the treated (polluted) group. This 0.6337% increase, while less pronounced than apoptosis, indicates that polluted soil exposure also contributes to cell necrosis. Overall, exposure to polluted soil (with chemical fertilizers and insecticides) led to a pronounced reduce in cell viability and a significant enhancement in apoptotic events (early and late stages), with a smaller but noticeable rise in necrotic cells. These findings emphasize the adverse effects of soil pollution on cellular homeostasis, highlighting the critical need for sustainable pest management practices to mitigate such impacts.

The comet assay results showed a dramatic increase in DNA damage in the treated group (Fig. 3f &g) compared to the control (Fig. 3e), with tail length rising from 1.428 μm to 7.992 μm, tail DNA percentage increasing from 1.480 to 5.237%, and tail moment escalating from 2.115 to 41.86 (Fig. 3h). The percentage of tailed cells also rose significantly from 1.683 to 17.00%, demonstrating substantial genotoxic effects induced by the treatment. increasing both early and late apoptosis (Fig. 3h). These findings suggest that the treatment predominantly induces apoptosis with a relatively minor contribution from necrotic cell death.

The observed increase in both early and late apoptosis, along with minimal necrotic cell death, supports the hypothesis that apoptosis is the primary mode of cell elimination in response to the treatment. This apoptosis-dominant mechanism may be attributed to DNA strand breaks triggering intrinsic apoptotic signaling cascades, ultimately resulting in cell cycle arrest and cell death.

### The level of stress markers in *P. bispinosus* after exposure to polluted soil

The biochemical assay results reveal significant alterations in the activities of antioxidant enzyme and the markers for oxidative stress in *P. bispinosus* larvae exposed to polluted soil compared to the control group (Fig. 3i). Malondialdehyde (MDA) levels, an indicator of lipid peroxidation and oxidative damage, were markedly elevated in the pollution-exposed larval group compared to the non-exposed larvae (control). While the control group ranged from 4.3 to 6.8 nmol/mg tissue, the treated group exhibited significantly higher levels, ranging from 11.6 to 13.2 nmol/mg tissue. SOD activity, a critical enzyme in neutralizing (ROS), was substantially reduced under soil pollution exposure. The control group showed activities ranging from 19.6 to 22.5 mU/mg protein, whereas the group exposed to soil pollution ranged from 8.16 to 9.4 mU/mg protein, highlighting a significant decline in antioxidant defense. CAT activity followed a similar trend, with the control group demonstrating higher levels (19.7–22.5 U/mg protein) compared to the treated group (10.3–12.4 U/mg protein). This reduction indicates impaired breakdown of hydrogen peroxide, a critical step in maintaining cellular redox balance. GSH levels, an essential antioxidant molecule, were significantly lower in the soil pollution-exposed larvae. Control group values ranged from 28.2 to 32.6 nmol/mg tissue, while treated larvae exhibited reduced levels between 18.05 and 22.5 nmol/mg tissue, highlighting depleted antioxidant reserves.

Glutathione Reductase GR activity, which regenerates reduced glutathione, also showed a marked decline. Control group activity ranged from 30.8 to 33.2 mU/mg protein, compared to 18.6 to 22.3 mU/mg protein in the group exposed to soil pollution. Ascorbate Peroxidase APOX activity was notably elevated in the treated group, with values ranging from 28.5 to 36.4 U/mg protein, compared to 13.6 to 17.5 U/mg protein in the control group. This increase may reflect a compensating response for oxidative stress.

Glutathione Peroxidase GPx activity also showed significant reductions under pollution exposure, with control group values between 18.2 and 21.4 mU/mg protein, compared to 7.6 to 11.3 mU/mg protein in the treated group. Overall, exposure to soil pollution caused a pronounced increase in oxidative stress markers (MDA and APOX), along with significant reductions in key antioxidant defenses (SOD, CAT, GSH, GR, and GPx). These changes suggest a substantial disruption of redox homeostasis, indicating that exposure to polluted soil induces oxidative stress and impairs the antioxidant system in *P. bispinosus* larvae.

The Cytochrome P450 assessment revealed a significant increase in enzymatic activity in the treated samples compared to the control (Fig. 3j). The mean Cytochrome P450 activity in the control group was 1.723, whereas the treated group exhibited a markedly elevated mean value of 5.490. This represents an approximately 3.19-fold increase in enzymatic activity following polluted soil exposure.

The substantial rise in Cytochrome P450 activity observed in the treated group suggests an adaptive metabolic response to soil pollution exposure, likely associated with detoxification processes and oxidative stress management. The enhanced enzyme levels may indicate an upregulation of the Cytochrome P450 system as part of the organism’s defense mechanisms to metabolize and eliminate toxic compounds introduced by soil pollution treatment.

Statistical analysis confirmed that the differences between treated and control groups were highly significant (*p* < 0.01), highlighting the potential of soil pollution exposure to induce metabolic stress and enzyme activation. These findings are consistent with previous studies demonstrating Cytochrome P450 induction as a biomarker of chemical stress, xenobiotic metabolism, and oxidative damage^36^. Further investigation is warranted to explore the molecular processes underlying this response and its implications for cellular detoxification and oxidative damage pathways. The acetylcholinesterase (AChE) activity assay revealed a significant reduction in enzymatic activity in the treated (polluted) group compared to the control group. The mean AChE activity in the control group was 0.7967, whereas the treated group exhibited a markedly lower mean value of 0.3667. This represents an approximate 54.0% decrease in enzymatic activity following soil pollution exposure.

The observed inhibition of AChE activity in the treated group indicates a direct neurotoxic effect of the pesticide residues, potentially through the irreversible binding of heavy metals, organophosphate or carbamate compounds to the enzyme’s active site. Such inhibition disrupts normal neurotransmission by preventing the breakdown of acetylcholine, leading to its accumulation at synapses. Statistical analysis confirmed that the reduction in AChE activity between the control and treated groups was highly significant (*p* < 0.01), indicating that soil pollution exposure exerts a pronounced inhibitory effect on cholinergic function (Fig. 3k).

### Micro- and ultrastructure analysis of *P. bispinosus* after exposure to soil pollution

The structure and ultrastructure of *P. bispinosus* larval midgut were prompted by our interest in understanding the impact of soil pollution owing to the use of pesticides and chemical fertilizers on the histological and physiological properties of the digestive tract of these soil-dwelling larvae and potential of *P. bispinosus* larvae as bioindicators of soil pollution.

#### Light microscopic view

**Fig. 4:**
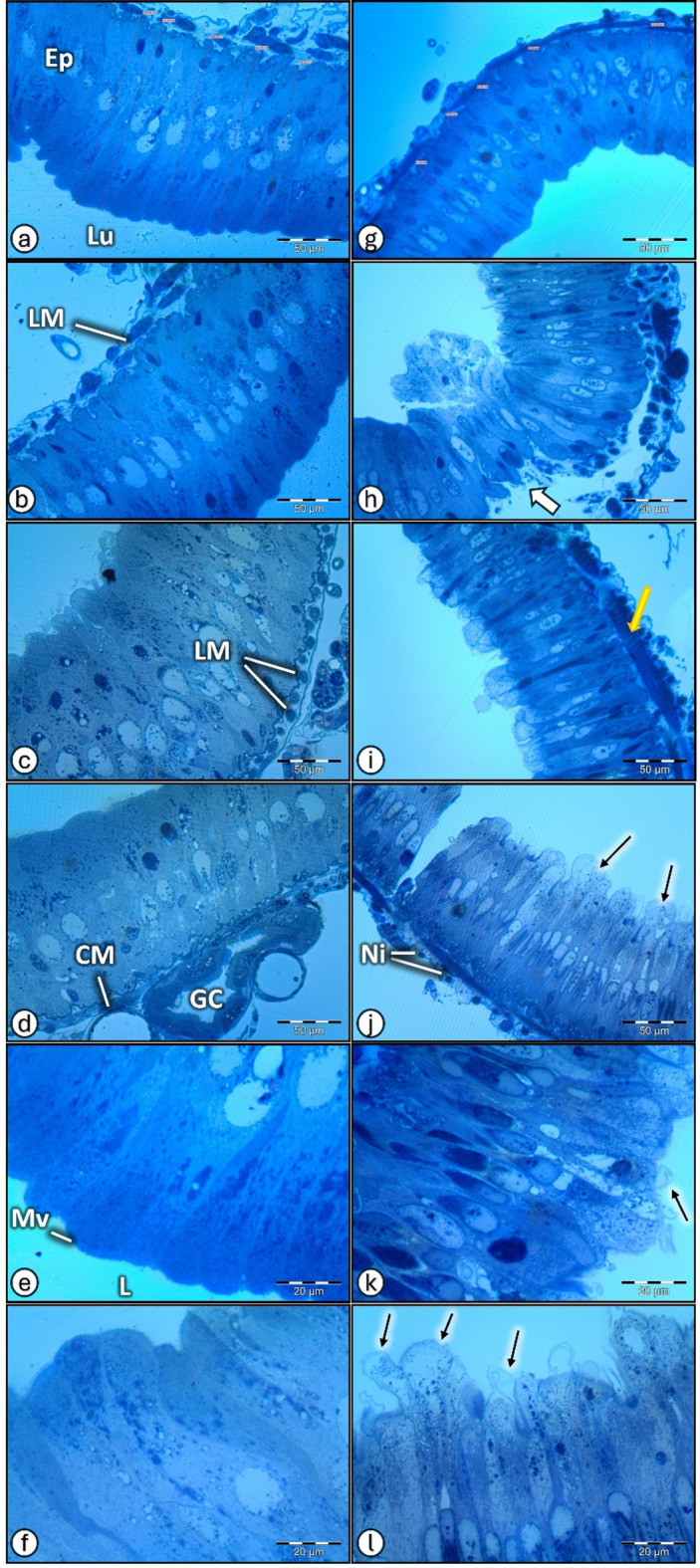
Light microscopic view of transverse-section of *P. algerinum* larval midgut from the selected sites. (**a**–**f**) A normal midgut of the control insect showing a normal appearance, (**a**) (X40) with broad epithelial layer. (**g**–**l**) Larval midgut of polluted insect showing appearance with abnormalities, (**g**) (X40) thinner epithelial layer. (**h**) thin part with separation of the epithelial layer from the muscular layer forming spaces (white arrow). (**j**–**l**) thin part with disruption, break off in epithelial layer, and rupture of microvilli. *Lu* lumen, *Ep* epithelium cells, *LM* Longitudinal muscles, *CM* circular muscles, *GC* Gastric caeca, *mv* microvilli, apical extrusions of digestive cells (arrows) towards midgut lumen Ni = nidi.

The transverse section of *P. bispinosus* larval midgut (Fig. 4) from the control insect showing a normal appearance of midgut with one-cell thick columnar or elongate epithelium enclosed by a sheath of circular and longitudinal muscles (Fig. 4a-f), with broad epithelial layer ranged from 135.32 to156.50 μm (X40) (Fig. 4a). The section of larval midgut from polluted site (Fig. 4g-l) showing abnormal appearance, (Fig. 4g) (X40) with thinner epithelial layer ranged from 93.00 to100.85 μm. The exposure to soil pollution damaged the midgut cells of *P. bispinosus* larvae and caused separation of epithelial layer from the muscle layer forming spaces (white arrow), also, some cell fragments with nucleus, were freed into the midgut lumen (Fig. 4h) with break off in the epithelial layer, disrupted epithelial layer, and rupture of microvilli (Fig. 4j, k &l).

#### Transmission electron micrographs

**Fig. 5:**
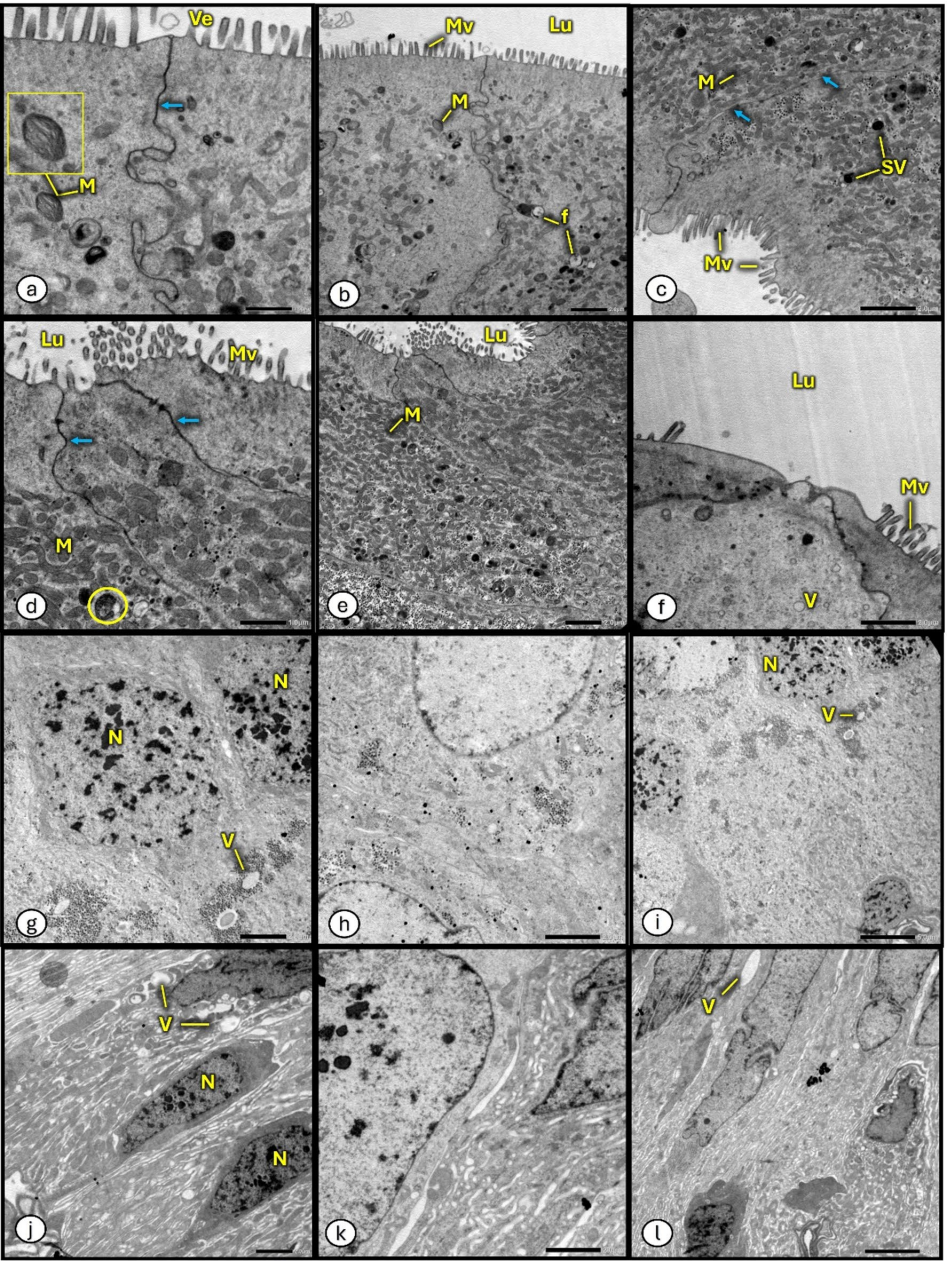
Electron micrographs of *P. bispinosus* larval midgut, collected from the selected sites. (**a**–**c**, **g**–**i**) are normal midgut of the control group. (**d**–**f**, **j**–**l**) are midgut of the treated (polluted) group. *N* nucleus, *Mv* microvilli, *V* vacuole, *Lu* lumen, *M* mitochondrion, *Ve* vesicle, *SV* secretory vesicle, *f* laminate sphere, (yellow circle) = nucleus with formation of condensed chromatin clusters, and (arrows) = Long septal junction between two non-vacuolated cells. [Scale bar = 1.0 μm in a, d. Scale bar = 2.0 μm in b, c, e, f, g, h, j, k. Scale bar = 5.0 μm in i and l]

Transmission electron micrographs of midgut of *P. bispinosus* larvae from the control site were illustrated in (Fig. 5a-c & g-i), midgut regions with well-developed microvilli, granules and mitochondria. After soil pollution exposure, the midgut of *P. bispinosus* larvae showed digestive cells with cytoplasm rich in granules, vacuoles and mitochondria with different shapes (Fig. 5d-f & j-l).

#### Scanning electron microscopy

**Fig. 6:**
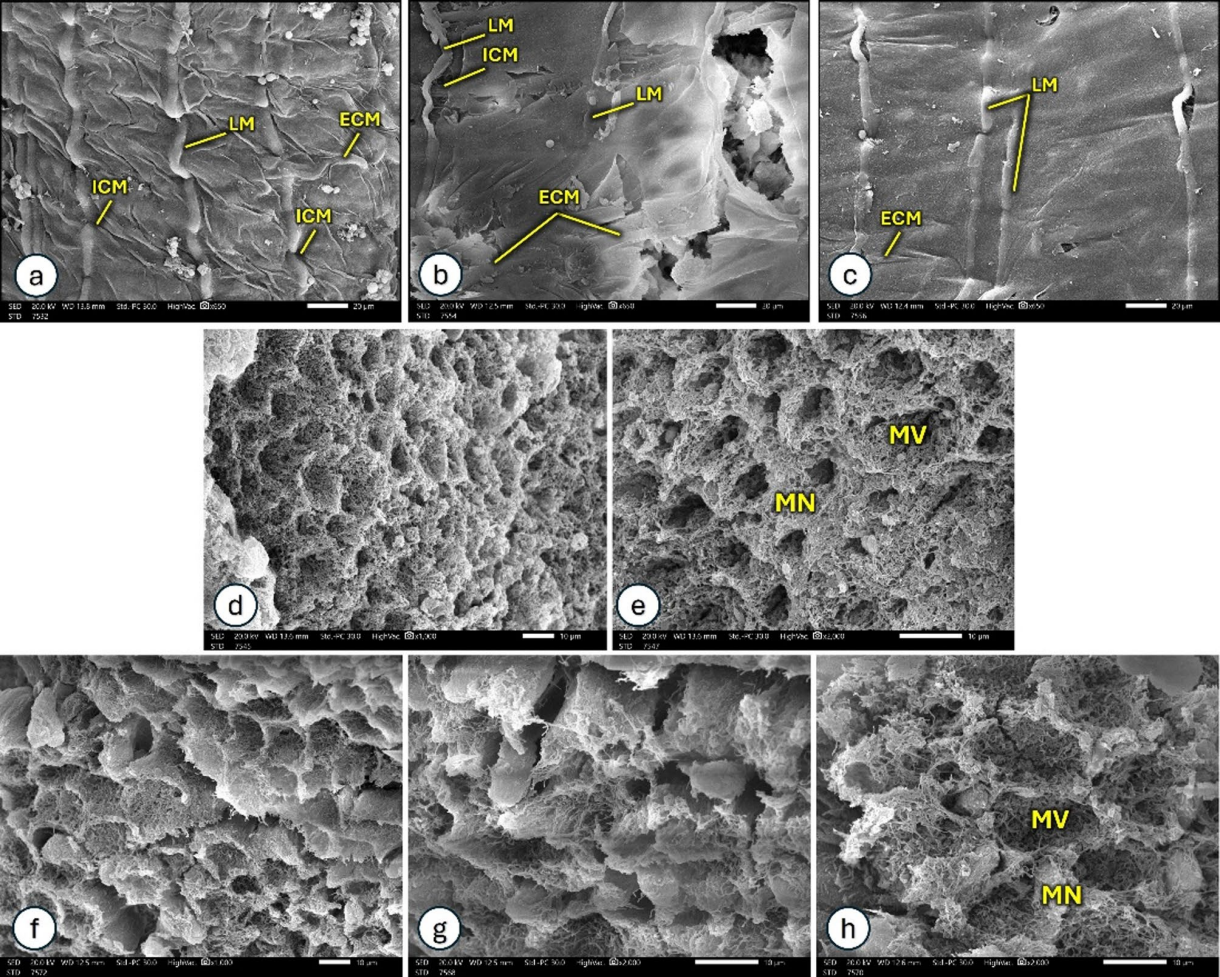
The muscle organization of larval midgut (**a**) in control group: bundles of longitudinal muscle (LM) and circular muscles which are organized in two layers, the external circular muscles (ECMs) and internal circular muscles (ICMs). (**b**, **c**) The larval midgut of polluted group. A view of the luminal midgut of *P. bispinosus* larvae, showed the microvilli-associate network. (**d**, **e**) The control group, the network strands are combined and form thick bundles (MN). Through some holes in the microvilli-associated network, the microvilli (MV) are visible. (**f**–**h**) The polluted group, the network bundles appear irregular, ragged and frayed.

The muscularis of the *P. bispinosus* larval midgut is arranged as a grid-like network of multiple longitudinally and circularly oriented bands, each composed of one or more muscle fibers (Fig. 6a). In many points, the circular and longitudinal bands visibly overlap at crossing intersections, with the longitudinal bands running externally to the circular ones. The circular muscles were organized into two layers, the external circular muscles (ECMs) and internal circular muscles (ICMs). The larval midgut muscularis of polluted group (Fig. 6b & c) have abnormal, irregular appearance and the muscles are mostly absent or undeveloped. The midgut wall tissues are torn in some parts.

A view of the midgut lumen of *P. bispinosus* larvae is presented in Fig. 6. Scanning electron microscopy showed the microvilli-associate network in control group (Fig. 6d &e), the network strands are combined and form thick bundles. The microvilli are visible through some holes in the microvilli-associated network. The network bundles in the polluted group appear irregular, ragged and frayed (Fig. 6f-h).

**Fig. 7:**
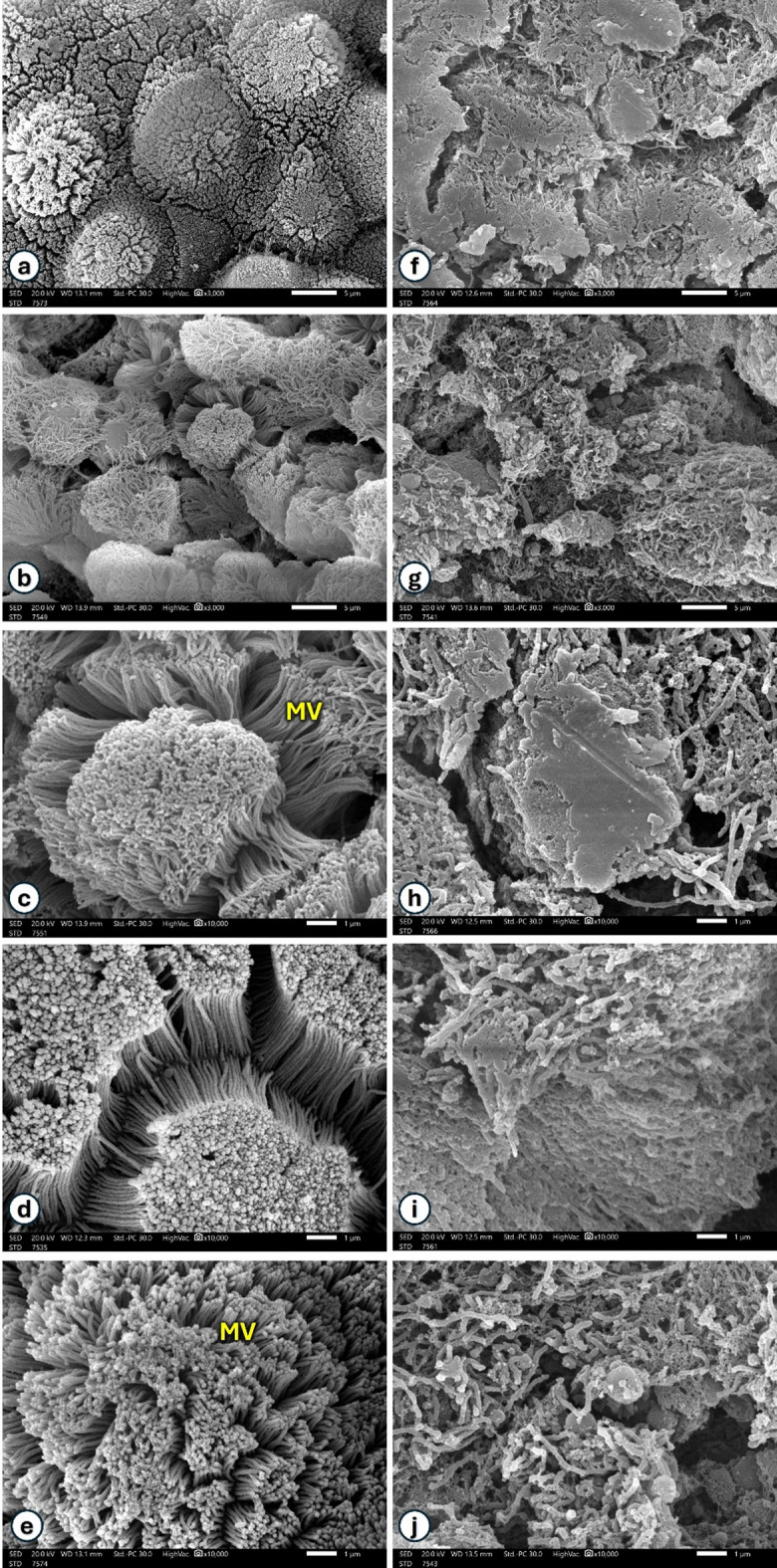
A view of the midgut lumen side of *P. bispinosus* larvae. Cells appear as round, regular microvilli mounds in control group (**a**, **b**) (Scale bar = 5 μm) and (**c**–**e**) (Scale bar = 1 μm). The polluted group (**f**, **g**) (Scale bar = 5 μm) and (**h**–**j**) (Scale bar = 1 μm), showed abnormal appaerance of midgut microvilli which appear to be lumpy, compacted, stuck together (**f**, **h** and **i**) and fused (**g**, **j**).

The midgut lumen side of *P. bispinosus* larvae was scanned with (Scale bar = 1&5 μm) and showed in Fig. 7. Cells appear as round, regular microvillated mounds in control group (Fig. 7a-e). The midgut microvilli showed abnormal appaerance in polluted group, microvilli appear to be lumpy, compacted, stuck together and fused (Fig. 7f-j).

## Discussion

The physiological, molecular, biochemical, and histological impacts on midgut tissues of *P. bispinosus* larvae caused by soil contaminated with heavy metals were determined. The findings of this study demonstrate a clear disruption of cellular homeostasis and integrity in *P. bispinosus* larvae exposed to a polluted soil. The significant reduction in cell viability, coupled with the marked increases in early and late apoptotic cells, underscores the cytotoxic effects of soil pollution exposure.

These results aligned with previous studies^37,38^, show that soil pollution with pesticide residues can induce oxidative stress, disrupt membrane integrity, and activate apoptotic pathways in insects.

The data further supports the hypothesis that organic environments provide a protective effect on cellular function, while pollution exposure compromises cellular integrity, potentially impacting the overall health and survival of *P. bispinosus* larvae.

The comet assay results revealed a significant elevation in DNA damage in the treated polluted group compared to the control, indicating the genotoxic potential of the treatment, underscoring the extent of DNA fragmentation induced by the treatment. These findings suggest that the treatment exerts potent genotoxic effects, which may activate DNA damage response pathways, leading to programmed cell death.

Our biochemical assays demonstrate significant changes in the markers of oxidative stress and activities of antioxidant enzymes. Lipid Peroxidation (MDA Levels), as measured by MDA, showed a marked increase in the treated group, which is consistent with findings in other studies that have shown elevated lipid peroxidation in organisms exposed to pesticides pollution^39,40^.

SOD is a primary defense against reactive oxygen species (ROS), and its inhibition in the treated group suggests an overwhelmed or inhibited antioxidant response due to increased ROS levels^41^. A significant decline in Superoxide Dismutase (SOD) activity was detected in the polluted soil-exposed larvae. This reduction in SOD activity aligns with findings in other studies showing diminished antioxidant activity following pesticide exposure^42,43^. Similarly, the activity of Catalase (CAT) was significantly reduced in the treated group. Catalase plays a crucial role in neutralizing hydrogen peroxide, a reactive oxygen species that can lead to DNA damage if not properly managed. The decreased CAT activity in this study mirrors previous research, where pesticide exposure led to the impairment of catalase activity in various organisms^44^. The Glutathione (GSH) levels were significantly lower in the treated larvae compared to the control, reflecting a depletion of one of the most important cellular antioxidants. Reduced GSH levels are frequently associated with oxidative stress, and our results are consistent with studies that have shown GSH depletion following pesticide exposure^44^. This reduction in GSH may compromise the larvae’s ability to detoxify ROS and maintain cellular redox homeostasis. Moreover, Glutathione Reductase (GR) activity was also significantly reduced in the treated group, suggesting impaired regeneration of reduced glutathione. The decrease in GR activity further supports the conclusion that pesticide exposure disrupts cellular antioxidant defense mechanisms^42^. Interestingly, ascorbate peroxidase plays a role in detoxifying hydrogen peroxide, and its elevated activity might reflect an attempt by the larvae to counterbalance the oxidative damage induced by soil pollution. This finding is consistent with studies that show increased antioxidant enzyme activities as a compensatory response to pesticide-induced oxidative stress^45^. Finally, Glutathione Peroxidase (GPx) activity was also significantly reduced in the treated larvae. Similar findings have been reported in other insect species exposed to pesticides^46^.

The significant increase in Cytochrome P450 activity observed in the treated group compared to the control group highlights the enzymatic induction triggered by soil pollution exposure. The 3.19-fold elevation in Cytochrome P450 activity suggests that soil pollution may have activated detoxification pathways, as this enzyme family plays a critical role in metabolizing xenobiotics and neutralizing toxic compounds. Similar findings have been reported in previous studies, where Cytochrome P450 upregulation was linked to oxidative stress and the metabolism of reactive intermediates^47^. This increase in activity could be a compensatory mechanism by the organism to manage the oxidative burden and eliminate toxic residues generated during pesticide degradation. Furthermore, the enhanced Cytochrome P450 activity supports its potential role as a biomarker for chemical pollution-induced metabolic stress. Elevated enzyme levels often correlate with increased reactive oxygen species (ROS) production, which may lead to cellular damage and oxidative stress-related disorders^48^.

The AChE activity in the treated group exhibited markedly lower value following soil pollution exposure compared to the control group. These results are consistent with previous studies that reported AChE inhibition as a biomarker of pesticide toxicity^36^. As Badiou et al., & Porrini et al.^49,50^, implies that AChE activity in live bees *Apis mellifera* may serve as a reliable biomarker to the insecticide exposure. AChE may conceivably be used as a biomarker of pollution by pesticide products and suggested for the monitoring of crucial circumstances that implicate pesticides in a particular areas, cause bee colonies to collapse and die, and are known to generate issues for the apiculture sector^51^.

The midgut epithelium is made up of a one-cell layer of various cell types, with columnar cells (those with a microvilli brush border) being the most frequent. The epithelium is additionally separated from the food bolus by a non-cellular film of semi-permeable peritrophic membrane. Finally, the alimentary canal exhibits the basal membrane, as well as the inner circular and outer longitudinal muscle layers^52^. The digestive tract most exposed to pollutants is midgut because it serves as an interface between the internal and exterior environments^53^. The midgut digests and absorbs harmful compounds, which may directly injure to the insect’s gut^54,55^. The digestive system may experience mitochondrial disruption, fragmented and disorganized microvilli, increased cytoplasmic vacuolization, brush boundary detachment or degradation, or enhanced cytoplasmic proteolysis^56^.

The morphology, structural and ultrastructural features of *P. bispinosus* larvae midgut was analyzed by light, scanning (SEM) and transmission (TEM) electron microscopy to assess the impact of soil pollution with heavy metals due to the use of insecticides and chemical fertilizers on larval midgut. The changes caused by exposure to polluted soil in the midgut histology indicate an injurious effect on the functions of midgut. These harmful effects were previously confirmed by Wu, et al.^57^ who highlighted that the ultrastructure of *Boettcherisca peregrina’s* midgut changed after being fed Cu and Cd. Where, the midgut becomes shorter, darker, and thicker, and different strumae appeared on the surface of the midgut. The midgut rough endoplasmic reticulum showed distention and vesiculation. Lysis, edema, and mitochondrial condensation were seen in the midgut. The microvilli in the midgut became shorter and disordered. Mineral spherites emerged in the insect’s midgut. Dabour, et al.^58^ found that honeybees fed a meal supplemented with sublethal doses of heavy metals, cadmium, and lead oxides, demonstrate cellular damage to the midgut tissue and destruction of the peritrophic membrane. The midgut cells of treated *P. bispinosus* larvae have cytoplasm rich in granules, vacuoles and mitochondria with different shapes. Lima et al.^59^, previously indicated that the pesticide (Abamectin) injured the midgut cells of *Anticarsia gemmatalis*, causing peritrophic matrix degeneration, disorganization of the epithelial striated border, accretion of mitochondria of various forms, increased autophagic properties, and cell fragments releasing into midgut lumen. Kheirallah and El-Samad^60^, studied the impact of heavy metals on *Trachyderma hispida* and *Blaps polycresta* (Or.: Coleoptera; Fam.: Tenebrionidae). Authors revealed that the deformation of the brush boundaries of the microvilli was the most common histological change identified as a result to heavy metal exposure. Ultrastructural changes comprised nuclear alteration of regenerating and digesting cells, lysis of the mitochondrial matrices, the appearance of electron dense vesicles, the existence of myelin figures, cytoplasmic vacuolation, and dilation of the endoplasmic reticulum (rough and smooth).

## Conclusion

The use of pesticides and mineral fertilizers increased heavy metal concentration in the soil and negatively impacted the agroecosystem. Relying on insects as a bioindicator has become a promising new issue concerning assessing environmental conservation. This study inspects the impacts of soil pollution on *P. bispinosus* larvae, and to prove that white grub beetles are promising bioindicators for soil pollution. The present findings highlight the need for adopting environmentally friendly pest management strategies and use of organic fertilizers instead of chemical mineral fertilizers to reduce the soil pollution stress (heavy metal accumulation) and consequently collateral damage to non-target organisms.

Our results concluded that scarab beetles *P. bispinosus* may efficiently be used as a natural bioindicator of the soil pollution.

## Data Availability

Data available from corresponding author on reasonable request.
